# Synthesis and Broad Antiviral Activity of Novel 2-aryl-isoindolin-1-ones towards Diverse Enterovirus A71 Clinical Isolates

**DOI:** 10.3390/molecules24050985

**Published:** 2019-03-11

**Authors:** Yixuan Wang, Huiqiang Wang, Xinbei Jiang, Zhi Jiang, Tingting Guo, Xingyue Ji, Yanping Li, Yuhuan Li, Zhuorong Li

**Affiliations:** 1 CAMS Key Laboratory of Antiviral Drug Research, Institute of Medicinal Biotechnology, Chinese Academy of Medical Sciences and Peking Union Medical College, 1 Tiantanxili, Beijing 100050, China; 18645148632@163.com (Y.W.); wanghuiqiang@imb.pumc.edu.cn (H.W.); Jiangxinbei2011@163.com (X.J.); jiangzhi450@163.com (Z.J.); guott2019@163.com (T.G.); jixingyue@imb.pumc.edu.cn (X.J.); lizhuorong@imb.pumc.edu.cn (Z.L.); 2 Beijing Key Laboratory of Antimicrobial Agents, Institute of Medicinal Biotechnology, Chinese Academy of Medical Sciences and Peking Union Medical College, 1 Tiantanxili, Beijing 100050, China

**Keywords:** 2-aryl-isoindolin-1-ones, enterovirus A71, synthesis, antiviral, structure activity relationship

## Abstract

Enterovirus 71 (EV-A71) is the main causative pathogen of childhood hand, foot and mouth disease. Effective medicine is currently unavailable for the treatment of this viral disease. Using the fragment-hopping strategy, a series of 2-aryl-isoindolin-1-one compounds were designed, synthesized and investigated for their in vitro antiviral activity towards multiple EV-A71 clinical isolates (H, BrCr, Shenzhen98, Jiangsu52) in Vero cell culture in this study. The structure–activity relationship (SAR) studies identified 2-phenyl-isoindolin-1-ones as a new potent chemotype with potent antiviral activity against EV-A71. Ten out of the 24 tested compounds showed significant antiviral activity (EC_50_ < 10 µM) towards four EV-A71 strains. Compounds **A3** and **A4** exhibited broad and potent antiviral activity with the 50% effective concentration (EC_50_) values in the range of 1.23–1.76 μM. Moreover, the selectivity indices of **A3** and **A4** were significantly higher than those of the reference compound, pirodavir. The western blotting experiment indicated that the viral VP1 was significantly decreased at both the protein and RNA level in a dose-dependent manner following treatment with compound **A3**. Moreover, compound **A3** inhibited the viral replication by acting on the virus entry stage. In summary, this study led to the discovery of 2-aryl-isoindolin-1-ones as a promising scaffold with potent anti-EV-A71 activities, which deserves further in-depth studies.

## 1. Introduction

Hand, foot and mouth disease (HFMD) is a seasonal epidemic infectious disease that mainly affects children under five years old [1,2]. Its common features include symptoms of pyrexia, vomiting, papulovesicular rash on the palms and soles, and oral ulcers in infected children. Enterovirus 71 (EV-A71), which was first isolated in 1969, is an enterovirus belonging to the Picornaradae family and is considered to be the main pathogen involved in HFMD [3,4]. Moreover, EV-A71 infection can cause severe nervous system diseases including encephalitis, myoclonic jerks, neurogenic pulmonary edema and even death [2,5]. EV-A71 infection has been a public health problem in China since the domestic outbreak in 2008, which led to nearly 490,000 reported cases [6]. More recently, cases of infection with EV-A71 have been increasing worldwide [7,8,9]. Although EV-A71 vaccines have been successfully developed and were approved in China in 2015, a protective immunological barrier has not been built due to low levels of vaccination in the population [10]. In addition, anti-EV-A71 drugs are under-developed and no specific antiviral agent is available on the market to treat EV-A71 infections.

**Figure 1:**
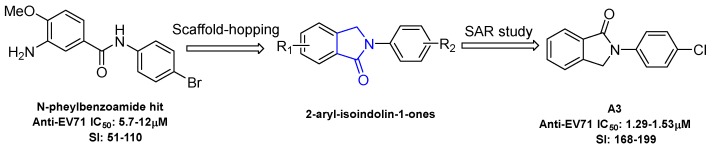
Structure modification strategy of anti-enterovirus 71 (EV71) compounds.

*N*-aryl benzamide was identified as a new broad antiviral chemotype in our previous research. The representative compound 3-amino-*N*-(4-bromophenyl)-4-methoxybenzamide (Figure 1) exerts moderate anti-EV-A71 activity against multiple virus strains [11]. However, the amide bond in *N*-aryl benzamide is liable to hydrolysis which may impose on the stability and the in vivo efficacy of the target compound. In order to mitigate this limitation, the incorporation of a cyclization constraint within this structure afforded a new series of 2-aryl-isoindolin-1-one compounds (Figure 1). The antiviral activity was evaluated in Vero cells infected with different EV-A71 clinical isolates. Although 2-Aryl isoindolin-1-ones with a variety of pharmacological effects, such as tumor necrosis factor (TNF)-α regulation, Alzheimer’s diagnostic probe, anti-diabetes effects and the promotion of bone growth have been reported [12,13,14], antiviral studies based on this type of scaffold have been less frequent. Herein, we describe preliminary SAR studies toward *N*-aryl isoindolin-1-one as a novel potent anti-EV-A71 chemotype. Several derivatives with favorable potency were obtained. Compounds **A3** and **A4** exhibited significant antiviral activity against four tested EV-A71 clinical isolates at a low micromole concentration. In addition, the preliminary mechanism of action studies revealed that compound **A3** played an antiviral role in the virus entry stage.

## 2. Results and Discussion

### 2.1. Chemistry

**Scheme 1:**
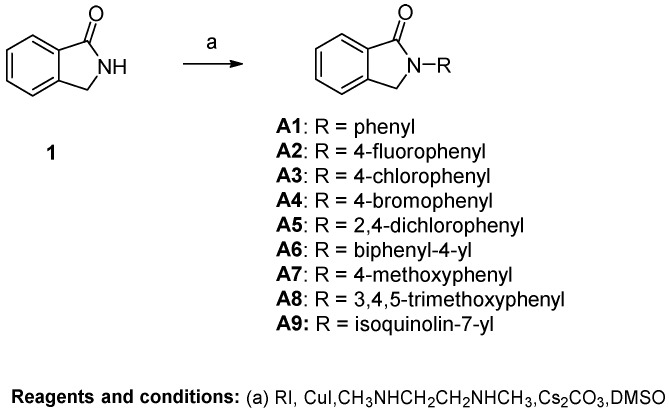
Preparation of *N*-substitutedphenyl-isoindolin-1-ones.

**Scheme 2:**
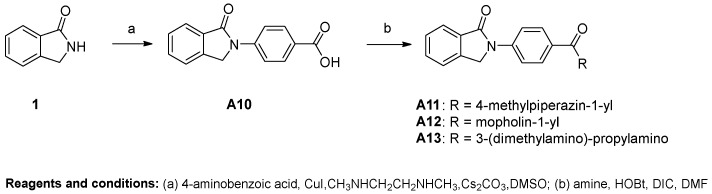
Preparation of *N*-(4-carbamoyl)phenyl-isoindolin-1-ones.

**Scheme 3:**
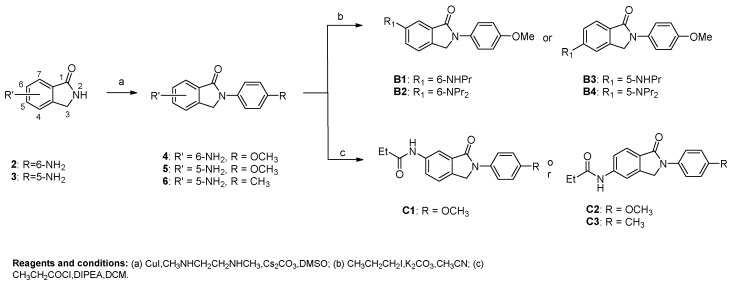
Preparation of 5 or 6-substituted *N*-phenyl-isoindolin-1-ones.

**Scheme 4:**
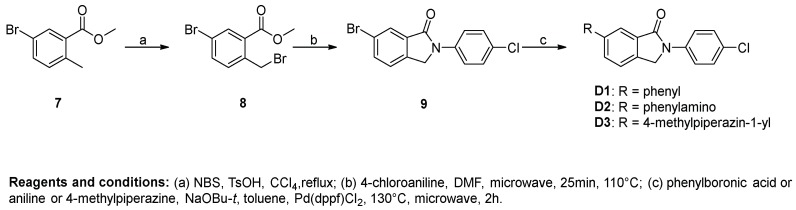
Preparation of 6-substituted *N*-(4-chlorophenyl)isoindolin-1-ones.

As shown in Scheme 1 and Scheme 2, different substituted iodobenzenes were chosen to react with commercially available isoindolin-1-one (**1**) to yield 2-aryl isoindolin-1-ones **A1**–**A10** by a modified Ullmann coupling reaction. Amides **A11**–**A13** were prepared by condensing **A10** with amines in the presence of 1-Hydroxybenzotriazole (HOBt) and *N*,*N*′-diisopropylcarbodiimide (DIC) in *N*,*N*-dimethylformide (DMF) (Scheme 2). As shown in Scheme 3, intermediates **4**–**6** were obtained by a similar method to that used for the synthesis of **A1**–**A10** with **2** or **3** as the starting materials. Then, **4** and **5** were alkylated with excessive iodopropane in the presence of anhydrous potassium carbonate in acetonitrile to afford compounds **B1**–**B4**. Compounds **4** and **5** were also acylated with propionyl chloride to quantitatively yield the target compounds **C1** and **C2**, respectively. Also, compound **C3** was synthesized by the propionylation of **6**. Furthermore, starting from methyl 5-bromo-2-methylbenzoate (**7**) in Scheme 4, intermediate **9** was subsequently obtained through bromination and a microwave-aided cyclization. Eventually, a phenyl, phenylamino or methylpiperazin group was introduced at the C6-position of isoindolinon **9** by a Buchwarld or Suzuki coupling reaction to afford the final compounds **D1**–**D3** with a yield of less than 40%.

### 2.2. In Vitro Antiviral Activity of N-aryl-isoindolin-1-ones against Multiple EV-A71 Clinical Isolates

An in-vitro antiviral assay was established using the Africa monkey kidney cell line Vero which was infected with different EV-A71 clinical isolates (BrCr, H, Shenzhen98 and Jiangsu52). A total of 24 synthetic compounds were initially screened in such an assay to determine their inhibitory effect on virus replication at 3-fold diluted concentrations serial (400~0.18 μM). The 50% effective concentration (EC_50_) of each compound was calculated according to its inhibitory rate values with the Reed–Muench method. The 50% cytotoxic concentration (CC_50_) was defined as the concentration that inhibited 50% cellular growth in comparison with the controls. The selectivity index (SI) was calculated as CC_50_/EC_50_. As shown in Table 1, the reference compound pirodavir, a potent picornavirus inhibitor, displayed potent inhibitory activity against four virus strains with EC_50_ values in the submicromolar range (EC_50_ = 0.38~0.76 µM) as well as high SI values (between 88 and 44) in this test model. Among the 24 tested compounds, 10 compounds (**A1**–**A5**, **A7**, **4**, **6**, **B1** and **D3**) showed significant antiviral activity (EC_50_ < 10 µM) and acceptable SI values towards four EV-A71 strains. Moreover, all EV-A71 strains showed similar sensitivity to the tested compounds. Importantly, compounds **A3** and **A4** possessed superior antiviral profiles (SI = 98–199) derived from their significantly lower cytotoxicity, although their EC_50_ values were about 2–4 fold higher (EC_50_ = 1.23~1.76 µM) than those of the reference compound, pirodavir. Generally, the EV-A71 H strain was less sensitive to most of the active compounds as compared to the other virus strains.

### 2.3. SAR Analysis

Most of the active compounds were 2-substitutedphenyl isoindolin-1-ones, whereas only a minority of the active compounds contained an amino group on the isoindolin-1-one moiety. Specifically, the replacement of phenyl (**A1**) with 4-halophenyl (**A2**–**A4**) increased the antiviral activity, whereas 4-methoxyphenyl substitution (**A5**) did not influence much of the antiviral activity against the four tested virus strains. Furthermore, 4-bromophenyl substitution (**A4**) provided comparable activity to 4-chlorophenyl substitution (**A3**), both of which were more potent than 4-fluorophenyl **A2**. Therefore, it can be concluded that lipophilicity is more important for potency than the electronic effect. Meanwhile, a halogen bond may exist within the interaction between compound **A3** or **A4** and their target. Although 3,4-dichlorophenyl substitution (**A5**) showed a similar potency as 4-chlorophenyl (**A3**), the former has a lower SI due to its increased cytotoxicity. The unfavorable meta-position substitution was further evidenced by the increased cytotoxicity of **A8** with multiple substitution as compared to **A7**, which has a single substituent at the para-position. The importance of lipophilicity at the C4-position of the 2-phenyl group for antiviral activity was further confirmed by the comparison between the halogen group (**A2**–**A5**) and isoquinoline (**A9**) or the carboxyl group (**A10**). The same trend was also observed for **5** and **6**. The potency was decreased when the carboxylic acid group of **A10** was further transformed into corresponding amide derivatives (**A11**–**A13**). Moreover, the potency was significantly compromised and even lost when the 2-phenyl group was replaced with a bulkier group such as biphenyl (**A6**). These results suggested that a steric limitation exists on the substituents at the pare-position of the 2-phenyl ring.

Compounds of the B, C and D series as well as compounds **4**–**6** were synthesized to probe the substitution effect on the isoindolin-1-one fragment. Firstly, the introduction of an amino group at the C6-position of isoindolin-1-one (**4**) slightly increased antiviral activity. On the contrary, the same substitution at the C5-position (**5**) decreased the activity as compared to **A7**. The presence of alkylamino group at the C6-postion (**B1**) led to a dramatically increased potency as compared to **B3** with the same substitution at the C5-position. The same trend was observed for the dialkylamino substituted compounds **B2** and **B4**. Of note, compounds with dialkylamino substitution on the isoindolin-1-one fragment consistently displayed weaker or even no activity compared to those with alkylamino group substitution. In addition, the higher potency of **B1** over **A7** illustrated the role of NH at the C6-position of isoindolin-1-one in antiviral activity. However, the acylation of C6-amino (**C1**) induced a several-fold decrease in potency in comparison with amino alkylation (**B1**). Among compounds **C1**–**C3**, only the C6-amide compound **C1** showed moderate activity, while the C5-amide compounds (**C2** and **C3**) completely lost their antiviral potency. These results reiterated the importance of the substitution position on isoindolin-1-one fragments. Encouraged by the good tolerance of 6-alkylamino substitution for anti-EV-A71 activity, we decided to further explore modification at that position to facilitate the SAR study. Unfortunately, the introduction of 6-phenyl (**D1**) resulted in a substantial loss in potency relative to that of **A3**. Similarly, 6-phenylamino substitution (**D2**) resulted in a 10-fold loss in activity against four in vitro models. Interestingly, 6-(4-methylpiperazin)-2-(4-chlorophenyl)-isoindolin-1-one (**D3**) showed comparable antiviral activity to that of **A3**. This is in contradiction to the former speculation about the importance of having the NH group at the C6-postion as a hydrogen-bond donor. A possible explanation for this disparity is that steric limitation at the C6-position plays a more crucial role in maintaining antiviral potency than the hydrogen bond.

Altogether, it appears that 2-substitutedphenyl-isoindolin-1-one is an important pharmacophore for anti-EV-A71 activity. A lipophilic group with size restriction was shown to be more favorable at the C4-position of the phenyl ring. A small amino group at the C6-position of the isoindolin-1-one moiety was also well tolerated.

### 2.4. Compound ***A3*** Inhibits EV-A71 Replication at both the RNA and Protein Levels

**Figure 2:**
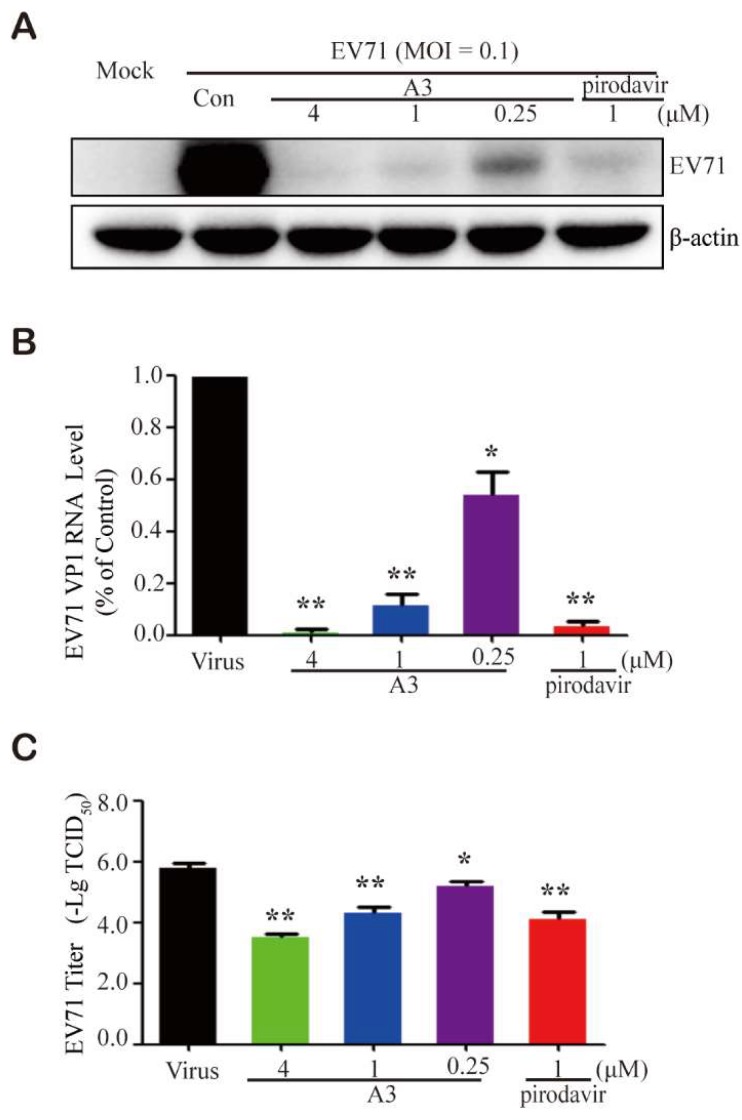
Compound **A3** inhibited EV-A71 replication in vitro. Vero cells (9 × 10^5^ cells/well) were plated into 6-well culture plates and infected with EV-A71 (multiplicity of infection, MOI = 0.1) for 1 h. The infected cells were then treated with the indicated concentrations of **A3** for 24 h. Intracellular viral VP1 protein (**A**) and RNA (**B**) were determined by Western blot and qRT-PCR assays, respectively. (**C**) The inhibition of a viral titer by **A3**. ** *p* < 0.01 * *p* < 0.05.

To further confirm the anti-EV-A71 activity, the expression levels of VP1 RNA and protein were determined. Vero cells were infected with the H strain of EV-A71 for 1 h followed by treatment with **A3** of various concentrations for another 24 h. As shown in Figure 2A, **A3** treatment decreased the levels of viral VP1 protein in a dose-dependent manner in vitro. Moreover, compound **A3** significantly reduced VP1 RNA expression level in a dose-dependent way in the reverse transcription-quantitative polymerase chain reaction (RT-qPCR) assay (Figure 2B). Meanwhile, the antiviral efficacy of **A3** was also tested using viral titer reduction assays. We observed a dose-dependent reduction in viral titers when the cells were treated with **A3** after EV-A71 infection (Figure 2C). Those results convincingly demonstrated that **A3** inhibited EV-A71 replication in vitro. 

### 2.5. Time-of-Addition Assay

**Figure 3:**
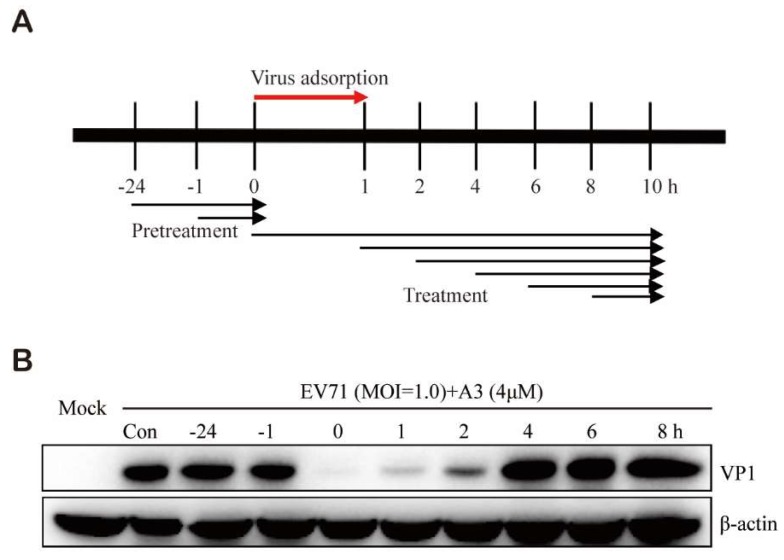
Time-of-addition analysis of Vero cells with compound **A3** treatment prior to, during or after EV-A71 infection. (**A**) Schematic illustration of experiment to determine which stage of EV-A71 viral cycle was inhibited by compound **A3** in Vero cells. (**B**) EV-A71 VP1 expression in Vero cells was significantly decreased when compound **A3** (4 μM) was administered at 0, 1 and 2 h after EV-A71 infection (MOI = 1.0) but not at the other time periods. For all experiments, the collected cells were washed with PBS after 10 h post viral infection, and viral VP1 expression was determined via Western blot assay.

In order to explore the inhibitory effect of compound **A3** on the EV-A71 viral life cycle, EV-A71 viral protein VP1 was measured via Western blot when Vero cells were treated with compound **A3** prior to, during, or after EV-A71 viral incubation (Figure 3). Vero cells pretreated with **A3** at 24 or 1 h prior to infection with EV-A71 did not display any resistance to infection by EV-A71. The maximum inhibitory activity was observed when the chemical was added during the inoculation of the virus. Meanwhile, compound **A3** treatment exerted significant efficacy when it was added at 1 or 2 h after EV-A71 infection, suggesting that **A3** may act on the early stage of viral life cycle. It is likely that compound **A3** inhibits the EV-A71 replication by acting on the virus entry stage.

## 3. Materials and Methods

### 3.1. Chemistry

All reagents and solvents used are commercially available and were used without further purification. ^1^H-NMR spectra were recorded in CDCl_3_ or DMSO-*d*_6_ on a Bruker Avance III 400MHz or a WNMR-1 500 MHz spectrometer (Wuhan Zhongke-Niujin, Wuhan, China). The chemical shift was reported in parts per million, relative to tetramethylsilane as the internal standard. High Resolution Mass Spectrum (HRMS) was recorded on a Triple TOF 5600+LC/MS/MS system (CADM-YQ-086) with an Electrospray Ionization (ESI) mass selective detector. The reaction was monitored through TLC Silica gel 60F254 Aluminium plate (Merck, 60F254D, Darmstadt, Germany). Flash chromatography was performed on a CombiflashRf 200 (Teledyne, Lincoln, NE, USA) with gel silica column.

General Procedure A for the Synthesis of Compounds A1~A12. Isoindolin-1-one (200 mg, 1.50 mmol) was dissolved in super-dry DMSO (4 mL), and Cs_2_CO_3_ (1215 mg, 3.75 mmol), CuI (58 mg, 0.30 mmol) and *N*^1^,*N*^2^-dimethylethane-1,2-diamine (27 mg, 33 μL, 0.30 mmol) were added to the solution. The resulting mixture was stirred at room temperature for 10 min, after which iodobenzene (2.25 mmol) was added. Then the mixture was heated to 120 °C. When TLC showed that isoindolin-1-one had been fully converted, the reaction was stopped. The mixture was extracted with ethyl acetate (20 mL) and H_2_O (10 mL). The water phase was re-extracted with ethyl acetate (20 mL). The organic layer was combined and washed with brine (10 mL). Then the solution was dried over anhydrous MgSO_4_, filtered and concentrated, and the crude residue was purified by flash chromatography over silica gel using CH_2_Cl_2_/CH_3_OH as the gradient elution to afford the title compounds.

*22-phenylisoindolin-1-one* (**A1**). Compound **A1** was synthesized from isoindolin-1-one and iodobenzene using general procedure A as a white solid. The yield was 50%. ^1^H-NMR (500 MHz, Chloroform-*d*) δ 7.98 (d, *J* = 7.4 Hz, 1H), 7.92 (d, *J* = 7.8 Hz, 2H), 7.64 (t, *J* = 7.5 Hz, 1H), 7.56 (d, *J* = 8.0 Hz, 2H), 7.49 (d, *J* = 8.0 Hz, 2H), 7.22 (d, *J* = 7.3 Hz, 1H), 4.91 (s, 2H). HRMS(ESI^+^) *m*/*z* calcd for C_14_H_12_NO [M + H]^+^ 210.0919, found 210.0921.

*2-(4-fluorophenyl)isoindolin-1-one* (**A2**). Compound **A2** was synthesized from isoindolin-1-one and 1-fluoro-4-iodobenzene using general procedure A as a yellow solid. The yield was 32%. ^1^H-NMR (500 MHz, DMSO-*d*_6_) δ 7.98 (dd, *J* = 8.8, 4.8 Hz, 2H), 7.84 (d, *J* = 7.5 Hz, 1H), 7.73 (s, 2H), 7.61 (d, *J* = 7.4 Hz, 1H), 7.35 (t, *J* = 8.7 Hz, 2H), 5.08 (s, 2H). HRMS(ESI^+^) *m*/*z* calcd for C_14_H_11_FNO [M + H]^+^ 228.0825, found 228.0827.

*2-(4-chlorophenyl)isoindolin-1-one* (**A3**). Compound **A3** was synthesized from isolindolin-1-one and 1-chloro-4-iodobenzene using general procedure A as a white solid. The yield was 49%. ^1^H-NMR (500 MHz, Chloroform-*d*) δ 7.97 (d, *J* = 7.7 Hz, 1H), 7.89 (d, *J* = 8.6 Hz, 2H), 7.66 (t, *J* = 7.4 Hz, 1H), 7.57 (d, *J* = 7.1 Hz, 2H), 7.44 (d, *J* = 8.6 Hz, 2H), 4.89 (s, 2H). HRMS(ESI^+^) *m*/*z* calcd for C_14_H_11_NOCl [M + H]^+^ 244.0529, found 244.0522.

*2-(4-bromophenyl)isoindolin-1-one* (**A4**). Compound **A4** was synthesized from isoindolin-1-one and 1-bromo-4-iodobenzene using general procedure A as a brown solid. The yield was 44%. ^1^H-NMR (500 MHz, DMSO-*d*_6_) δ 7.93 (d, *J* = 8.6 Hz, 2H), 7.83 (d, *J* = 7.6 Hz, 1H), 7.75–7.62 (m, 4H), 7.58 (t, *J* = 7.3 Hz, 1H), 5.06 (s, 2H). HRMS(ESI^+^) *m*/*z* calcd for C_14_H_11_BrNO [M + H]^+^ 288.0024, found 288.0026.

*2-(2,4-dichlorophenyl)isoindolin-1-one* (**A5**). Compound **A5** was synthesized from isolindolin-1-one and 2,4-dichloro-1-iodobenzene using general procedure A as a white solid. The yield was 48%. ^1^H-NMR (500 MHz, Chloroform-*d*) δ 8.01 (d, *J* = 7.6 Hz, 1H), 7.68 (t, *J* = 7.5 Hz, 1H), 7.58 (dd, *J* = 12.0, 5.9 Hz, 3H), 7.43 (t, *J* = 6.5 Hz, 2H), 4.85 (s, 2H). HRMS(ESI^+^) *m*/*z* calcd for C_14_H_10_NOCl_2_ [M + H]^+^ 278.0139, found 278.0132.

*2-([1,1’-biphenyl]-4-yl)isoindolin-1-one* (**A6**). Compound **A6** was synthesized from isoindolin-1-one and 4-iodo-1,1’-biphenyl using general procedure A as a yellow solid. The yield was 26%. ^1^H-NMR (500 MHz, DMSO-*d*_6_) δ 8.05 (d, *J* = 8.3 Hz, 2H), 7.84 (d, *J* = 7.6 Hz, 1H), 7.80 (d, *J* = 8.3 Hz, 2H), 7.73 (t, *J* = 5.6 Hz, 4H), 7.60 (d, *J* = 7.1 Hz, 1H), 7.51 (t, *J* = 7.6 Hz, 2H), 7.39 (t, *J* = 7.7 Hz, 1H), 5.12 (s, 2H). HRMS(ESI^+^) *m*/*z* calcd for C_20_H_16_NO [M + H]^+^ 286.1232, found 286.1220.

*2-(4-methoxyphenyl)isoindolin-1-one* (**A7**). Compound **A7** was synthesized from isolindolin-1-one and 1-iodo-4-methoxybenzene using general procedure A as a white solid. The yield was 53%. ^1^H-NMR (500 MHz, Chloroform-*d*) δ 7.96 (d, *J* = 7.6 Hz, 1H), 7.78 (d, *J* = 8.4 Hz, 2H), 7.62 (t, *J* = 7.4 Hz, 1H), 7.54 (s, 2H), 7.01 (d, *J* = 8.5 Hz, 2H), 4.85 (s, 2H), 3.87 (s, 3H). HRMS(ESI^+^) *m*/*z* calcd for C_15_H_14_NO_2_ [M + H]^+^ 240.1025, found 240.1020.

*2-(3,4,5-trimethoxyphenyl)isoindolin-1-one* (**A8**). Compound **A8** was synthesized from isoindolin-1-one and 3,4,5-trimethoxy-1-iodobenzene using general procedure A as a yellow solid. The yield was 37%. ^1^H-NMR (500 MHz, DMSO-*d*_6_) δ 7.77 (d, *J* = 7.6 Hz, 1H), 7.69 (dd, *J* = 6.7, 1.0 Hz, 2H), 7.60–7.51 (m, 1H), 7.28 (s, 2H), 5.05 (s, 2H), 3.83 (s, 6H), 3.67 (s, 3H). HRMS(ESI^+^) *m*/*z* calcd for C_17_H_18_NO_4_ [M + H]^+^ 300.1236, found 300.1222.

*2-(isoquinolin-7-yl)isoindolin-1-one* (**A9**). Compound **A9** was synthesized from isoindolin-1-one and 7-iodoisoquinoline using general procedure A as a yellow solid. The yield was 48%. ^1^H-NMR (500 MHz, Chloroform-*d*) δ 9.34 (s, 1H), 8.56 (d, *J* = 9.0 Hz, 2H), 8.32 (s, 1H), 8.01 (d, *J* = 7.6 Hz, 1H), 7.94 (d, *J* = 9.1 Hz, 1H), 7.69 (s, 2H), 7.66–7.50 (m, 2H), 5.05 (s, 2H). HRMS(ESI^+^) *m*/*z* calcd for C_17_H_13_N_2_O [M + H]^+^ 261.1028, found 261.1034.

*4-(1-oxoisoindolin-2-yl)benzoic acid* (**A10**). Compound **A10** was synthesized from isoindolin-1-one and 4-iodobenzoic acid using general procedure A as a white solid. The yield was 31%. ^1^H-NMR (500 MHz, DMSO-*d*_6_) δ 12.87 (s, 1H), 8.11–8.05 (m, 1H), 8.02 (dd, *J* = 9.1, 2.2 Hz, 1H), 7.83 (d, *J* = 7.6 Hz, 1H), 7.75–7.67 (m, 1H), 7.59–7.54 (m, 0H), 5.09 (s, 1H). HRMS(ESI^+^) *m*/*z* calcd for C_15_H_12_NO_3_ [M + H]^+^ 254.0817, found 254.0805

General Procedure for the Synthesis of Compounds **A11**–**A13**. Compound **A10** (100 mg, 0.3 mmol) was dissolved in DMF (3 mL). HOBt (69 mg, 0.4 mmol) and DIC (80 μL, 0.4 mmol) were added to the solution and the resulting solution was stirred for 20 min at room temperature. Amine was added into the resulting mixture and stirred at room temperature overnight. When TLC showed that the reaction was complete, the mixture was extracted with EA and H_2_O. The water phase was re-extracted with EA_._ The organic layer was combined and washed with brine (10 mL). Then the solution was dried over anhydrous MgSO_4_, filtered and concentrated, and the crude residue was purified by flash chromatography over silica gel using CH_2_Cl_2_/CH_3_OH as the gradient elution to afford the title compounds.

*2-(4-(4-methylpiperazine-1-carbonyl)phenyl)isoindolin-1-one* (**A11**). Compound **A11** was prepared from **A10** and 1-methypiperazine using the above procedure as a white solid. The yield was 61%. ^1^H-NMR (500 MHz, DMSO-*d*_6_) δ 8.03–7.98 (m, 2H), 7.82 (d, *J* = 7.6 Hz, 1H), 7.74–7.67 (m, 2H), 7.59–7.54 (m, 1H), 7.52–7.47 (m, 2H), 5.07 (s, 2H), 3.45 (dd, *J* = 59.5, 52.5 Hz, 4H), 2.35 (d, *J* = 16.8 Hz, 4H), 2.20 (s, 3H). HRMS(ESI^+^) *m*/*z* calcd for C_20_H_22_N_3_O_2_ [M + H]^+^ 336.1712, found 336.1697.

*2-(4-(morpholin-4-carbonyl)phenyl)isoindolin-1-one* (**A12**). Compound **A12** was prepared from **A10** and morpholine using the above procedure as a white solid. The yield was 65%. ^1^H-NMR (500 MHz, DMSO-*d*_6_) δ 8.01 (d, *J* = 8.7 Hz, 2H), 7.82 (d, *J* = 7.6 Hz, 1H), 7.76–7.65 (m, 2H), 7.61–7.47 (m, 3H), 5.08 (s, 2H), 3.62 (s, 8H). HRMS(ESI^+^) *m*/*z* calcd for C_19_H_19_N_2_O_3_ [M + H]^+^ 323.1396, found 323.1383.

*N-(3-(dimethylamino)propyl)-N-methyl-4-(1-oxoisoindolin-2-yl)benzamide* (**A13**). Compound **A13** was prepared from **A10** and 3-dimethylamino-propylamine using the above procedure as a shallow yellow solid. The yield was 40%. ^1^H-NMR (500 MHz, DMSO-*d*_6_) δ 8.54 (t, *J* = 5.5 Hz, 1H), 8.02 (d, *J* = 8.9 Hz, 2H), 7.93 (d, *J* = 8.9 Hz, 2H), 7.81 (d, *J* = 7.6 Hz, 1H), 7.76–7.65 (m, 2H), 7.61–7.52 (m, 1H), 5.08 (s, 2H), 3.30 (dd, *J* = 12.8, 6.8 Hz, 3H), 2.41 (t, *J* = 7.1 Hz, 2H), 2.25 (s, 6H), 1.75–1.63 (m, 2H). HRMS(ESI^+^) *m*/*z* calcd for C_20_H_24_N_3_O_2_ [M + H]^+^ 338.1869, found 338.1852.

*6-amino-2-(4-methoxyphenyl)isoindolin-1-one* (**4**). Compound **4** was synthesized from 6-aminoisoindolin-1-one **2** and 1-iodo-4-methoxybenzene using general procedure A as a white solid. The yield was 56.97%. ^1^H-NMR (400 MHz, DMSO-*d*_6_) δ 7.80–7.73 (m, 2H), 7.26 (d, *J* = 8.1 Hz, 1H), 7.03–6.95 (m, 2H), 6.93–6.82 (m, 2H), 5.38 (s, 2H), 4.77 (s, 2H), 3.77 (s, 3H), 2.55 (s, 7H). HRMS(ESI^+^) *m*/*z* calcd for C_15_H_15_N_2_O_2_ [M + H]^+^ 255.1134, found 255.1140.

*5-amino-2-(4-methoxyphenyl)isoindolin-1-one* (**5**). Compound **5** was synthesized from 5-aminoisolindolin-1-one **3** and 1-iodo-4-methoxybenzene using general procedure A as a yellow solid. The yield was 51.54%. ^1^H-NMR (500 MHz, DMSO-*d*_6_) δ 7.77 (d, *J* = 8.6 Hz, 2H), 7.42 (d, *J* = 8.1 Hz, 1H), 6.99 (d, *J* = 8.5 Hz, 2H), 6.87–6.50 (m, 2H), 5.92 (s, 2H), 4.78 (s, 2H), 3.78 (s, 3H). HRMS(ESI^+^) *m*/*z* calcd for C_15_H_15_N_2_O_2_ [M + H]^+^ 255.1134, found 255.1124.

*5-amino-2-(p-tolyl)isoindolin-1-one* (**6**). Compound **6** was synthesized from 5-aminoisoindolin-1-one **3** and 4-iodotoluene using general procedure A as a yellow solid. The yield was 45.69%. ^1^H-NMR (400 MHz, DMSO-*d*_6_) δ 7.74 (dd, *J* = 8.9, 2.4 Hz, 2H), 7.39 (d, *J* = 8.1 Hz, 1H), 7.26–7.12 (m, 2H), 6.65 (dd, *J* = 10.2, 2.1 Hz, 2H), 5.93 (s, 2H), 4.77 (s, 2H), 2.29 (s, 3H). HRMS(ESI^+^) *m*/*z* calcd for C_15_H_15_N_2_O [M + H]^+^ 239.1184, found 239.1185.

General Procedure B for the Synthesis of Compounds **B1**–**B4**. 6-amino-2-(4-methoxyphenyl)isoindolin-1-one **4** (300 mg, 1.18 mmol) was dissolved in superdry CH_3_CN (5 mL), and K_2_CO_3_ (449 mg, 3.54 mmol) was added to the solution. The resulting mixture was stirred at room temperature, after which 1-iodopropane (2.25 mmol) was added. When TLC showed that 6-amino-2-(4-methoxyphenyl)isoindolin-1-one was fully converted, the reaction was stopped. The mixture was filtered and the filtrate was evaporated in vacuo. The residue was dissolved in CH_2_Cl_2_ and then washed with H_2_O. The water phase was re-extracted with CH_2_Cl_2._ The organic layer was combined and washed with brine (10 mL). Then the solution was dried over anhydrous MgSO_4_, filtered and concentrated, and the crude residue was purified by flash chromatography over silica gel using CH_2_Cl_2_/CH_3_OH as the gradient elution to afford 2-(4-methoxyphenyl)-6-(propylamino)isoindolin-1-one (**B1**) and 6-(dipropylamino)-2-(4-methoxyphenyl)isoindolin-1-one (**B2**) as a yellow solid with yields of 32% and 26% for **B1** and **B2**, respectively.

Compound **B1** ^1^H-NMR (500 MHz, Chloroform-*d*) δ 7.75 (d, *J* = 8.5 Hz, 2H), 7.41 (d, *J* = 7.4 Hz, 2H), 7.22 (s, 1H), 7.00 (d, *J* = 8.5 Hz, 2H), 4.77 (s, 2H), 3.88 (s, 3H), 3.25 (t, *J* = 7.7 Hz, 2H), 1.81 (q, *J* = 7.6 Hz, 2H), 1.07 (t, *J* = 7.4 Hz, 3H). HRMS(ESI^+^) *m*/*z* calcd for C_18_H_21_N_2_O_2_ [M + H]^+^ 297.1603, found 297.1611.

Compound **B2** ^1^H-NMR (500 MHz, Chloroform-*d*) δ 7.77 (d, *J* = 8.5 Hz, 2H), 7.16 (s, 1H), 7.01 (d, *J* = 8.6 Hz, 2H), 6.90 (s, 1H), 4.87–4.66 (m, 2H), 3.87 (s, 3H), 3.35 (s, 4H), 1.68 (s, 4H), 0.98 (t, *J* = 7.4 Hz, 6H). HRMS(ESI^+^) *m*/*z* calcd for C_21_H_27_N_2_O_2_ [M + H]^+^ 339.2073, found 339.2055.

2-(4-methoxyphenyl)-5-(propylamino)isoindolin-1-one (**B3**) and 5-(dipropylamino)-2-(4-methoxyphenyl)isoindolin-1-one (**B4**) were synthesized from 5-amino-2-(4-methoxyphenyl)isoindolin-1-one (**5**) and 1-iodopropane using general procedure B, which were both yellow solids. The yields were 34% and 28% for **B3** and **B4**, respectively.

Compound **B3** ^1^H-NMR (500 MHz, Chloroform-*d*) δ 7.74 (dd, *J* = 14.5, 8.5 Hz, 3H), 6.99 (d, *J* = 8.6 Hz, 2H), 6.71 (d, *J* = 24.0 Hz, 2H), 4.75 (s, 2H), 3.87 (s, 3H), 3.21 (t, *J* = 7.1 Hz, 2H), 1.75 (q, *J* = 7.1 Hz, 2H), 1.07 (t, *J* = 7.4 Hz, 3H).

Compound **B4** ^1^H-NMR (500 MHz, Chloroform-*d*) δ 7.75 (dd, *J* = 13.3, 8.8 Hz, 3H), 6.99 (d, *J* = 8.8 Hz, 2H), 6.75 (d, *J* = 8.6 Hz, 1H), 6.66 (s, 1H), 4.76 (s, 2H), 3.87 (s, 3H), 3.37 (t, *J* = 7.8 Hz, 4H), 1.71 (h, *J* = 7.5 Hz, 4H), 1.01 (t, *J* = 7.4 Hz, 6H). HRMS(ESI^+^) *m*/*z* calcd for C_21_H_27_N_2_O_2_ [M + H]^+^ 339.2073, found 339.2076.

General Procedure C for the Synthesis of Compounds **C1**–**C3**. 5-amino-2-(*p*-tolyl)isoindolin-1-one **6** (70 mg, 0.29 mmol) was dissolved in CH_2_Cl_2_ (3 mL), and DIPEA (154 μL, 0.87 mmol) was added to the solution. Propionyl chloride was dissolved in CH_2_Cl_2_ (1 mL) and transferred to a drip funnel, and dropped into the flask, at a rate of about 1–2 drop(s) per second. After completion of the addition, the mixture was stirred at room temperature. When TLC showed that 6-amino-2-(*p*-tolyl)isoindolin-1-one had been fully converted, the reaction was stopped. The mixture was extracted with H_2_O (3 mL). The water phase was re-extracted with CH_2_Cl_2_ (10 mL). The organic layer was combined and washed with brine (10 mL). Then the solution was dried over anhydrous MgSO_4_, filtered and concentrated, and the crude residue was purified by flash chromatography over silica gel using CH_2_Cl_2_/CH_3_OH as the gradient elution to afford compound *N*-(1-oxo-2-(*p*-tolyl)isoindolin-5-yl)propionamide (**C3**) as a yellow solid. The yield was 46%. ^1^H-NMR (500 MHz, DMSO-*d*_6_) δ 10.27 (s, 1H), 8.07 (s, 1H), 7.80 (d, *J* = 7.9 Hz, 2H), 7.71 (d, *J* = 8.3 Hz, 1H), 7.63 (d, *J* = 8.5 Hz, 1H), 7.26 (d, *J* = 8.0 Hz, 2H), 4.97 (s, 2H), 2.41 (q, *J* = 7.5 Hz, 2H), 2.33 (s, 3H), 1.14 (t, *J* = 7.5 Hz, 3H). HRMS(ESI^+^) *m*/*z* calcd for C_18_H_19_N_2_O_2_ [M + H]^+^ 295.1447, found 295.1433.

*N-(2-(4-methoxyphenyl)-3-oxoisoindolin-5-yl)propionamide* (**C1**). Compound **C1** was synthesized from 6-amino-2-(4-methoxyphenyl)isoindolin-1-one (**4**) and propionyl chloride using general procedure C as a yellow solid. The yield was 49%. ^1^H-NMR (500 MHz, Chloroform-*d*) δ 8.22 (s, 1H), 7.85 (d, *J* = 8.1 Hz, 1H), 7.75 (d, *J* = 8.6 Hz, 2H), 7.63 (s, 1H), 7.00 (d, *J* = 8.5 Hz, 2H), 4.83 (s, 2H), 3.87 (s, 3H), 2.77–2.30 (m, 2H), 1.31 (t, *J* = 7.5 Hz, 3H). HRMS(ESI^+^) calcd for C_18_H_19_N_2_O_3_ [M + H]^+^ 311.1396, found 311.1386.

*N-(2-(4-methoxyphenyl)-1-oxoisoindolin-5-yl)propionamide* (**C2**). Compound **C2** was synthesized from 5-amino-2-(4-methoxyphenyl)isoindolin-1-one (**5**) and propionyl chloride using general procedure C as a yellow solid. The yield was 50%. ^1^H-NMR (400 MHz, DMSO-*d*_6_) δ 10.24 (s, 1H), 8.04 (d, *J* = 1.6 Hz, 1H), 7.82–7.75 (m, 2H), 7.68 (d, *J* = 8.2 Hz, 2H), 7.61 (dd, *J* = 8.3, 1.7 Hz, 1H), 7.04–6.97 (m, 2H), 4.94 (s, 2H), 3.77 (s, 3H), 2.39 (q, *J* = 7.5 Hz, 2H), 1.11 (t, *J* = 7.5 Hz, 3H). HRMS(ESI^+^) calcd for C_18_H_19_N_2_O_3_ [M + H]^+^ 311.1396, found 311.1382.

Procedure D for the synthesis of compounds **D1**–**D3**. Methyl 5-bromo-2-methylbenzoate (2.29 g, 10 mmol) was added to a flask containing 20 mL CCl_4_, and then *N*-bromosuccinimide (1.78 g, 10 mmol) and TsOH (172 mg, 1 mmol) were added. The mixture was refluxed until methyl 5-bromo-2-methylbenzoate was fully converted. The solution was washed with saturated NaHCO_3_ solution, water and brine successively. The organic phase was evaporated under vacuum to afford the crude product methyl 5-bromo-2-(bromomethyl)benzoate (**9**).

A 10 mL microwave vial was charged with 4-chloroaniline (638 mg, 5 mmol), methyl 5-bromo-2-(bromomethyl)benzoate (1.57 g, 5.5 mmol), and DMF (4 mL). The vial was sealed and microwaved at 110 °C for 25 min. The reaction mixture was diluted with MeOH (50 mL) and an off-white precipitate was formed, which was filtered and dried under vacuum to give **10** (1.19 g, 73.78%) as a white solid. ^1^H-NMR (500 MHz, DMSO-*d_6_*) δ 7.96 (d, *J* = 8.9 Hz, 3H), 7.90 (d, *J* = 8.1 Hz, 1H), 7.68 (d, *J* = 8.1 Hz, 1H), 7.54 (d, *J* = 8.4 Hz, 2H), 5.03 (s, 2H).

A microwave vial was charged with **10** (97 mg, 0.30 mmol), phenylboronic acid (41 mg, 0.33 mmol), NaOBu-*t* (58 mg, 0.60 mmol), DMSO (2 mL), and H_2_O (0.3 mL). The solution was degassed with nitrogen for 10 min, and Pd(dppf)Cl_2_ (12 mg, 0.015 mmol) was added. The vial was sealed and microwaved at 130 °C for 2 h. The reaction mixture was poured into EtOAc/H_2_O and the organic layer was separated and subsequently passed through a plug of florisil. The obtained organic phase was concentrated into a residue, which was triturated with MeOH, filtered, and dried under vacuum to afford **D1** (43 mg, 44.82%) as a white solid. ^1^H-NMR (500 MHz, DMSO-*d_6_*) δ 8.05–7.98 (m, 4H), 7.80 (d, *J* = 7.3 Hz, 3H), 7.58–7.50 (m, 4H), 7.45 (t, *J* = 7.3 Hz, 1H), 5.11 (s, 2H).

Compound **D2** was synthesized using **10** and aniline using the same method of synthesis as that of **D1**. **D2** was purified by flash chromatography to afford a yellow solid. The yield was 20%. ^1^H-NMR (400 MHz, DMSO-*d*_6_) δ 8.46 (s, 1H), 8.00–7.89 (m, 2H), 7.51 (dd, *J* = 8.5, 4.1 Hz, 3H), 7.45–7.24 (m, 4H), 7.14 (d, *J* = 7.9 Hz, 2H), 6.91 (t, *J* = 7.3 Hz, 1H), 4.93 (s, 2H).

Compound **D3** was synthesized using **10** and 4-methylpiperazine using the same method of synthesis as that of **D1**. **D3** was purified by flash chromatography to afford a pale yellow solid. The yield was 22%. ^1^H-NMR (500 MHz, DMSO-*d*_6_) δ 7.96 (d, *J* = 8.9 Hz, 3H), 7.90 (d, *J* = 8.1 Hz, 1H), 7.68 (d, *J* = 8.1 Hz, 1H), 7.54 (d, *J* = 8.4 Hz, 2H), 5.03 (s, 2H).

### 3.2. Cells and Viruses

African green monkey kidney (Vero) cells were purchased from the American Type Culture Collection (ATCC) and were cultured in minimum essential medium (MEM) supplemented with 10% fetal bovine serum (FBS) (GIBCO) and antibiotics (100 U/mL penicillin and 100 mg/mL streptomycin) at 37 °C in a 5% CO_2_ incubator.

EV-A71 strain ShenZhen98 (SZ98) was isolated from the throat swab sample of an HFMD case which occurred in 1998 in China. This was kindly provided by Dr. Qi Jin, Institute of Pathogen Biology, Chinese Academy of Medical Science and Peking Union Medical School, Beijing, China. EV-A71 strains BrCr (VR-1775) and H (VR-1432) were purchased from the ATCC. The EV-A71 strain JS52 was a kind gift from Dr. Xiangzhong Ye, Beijing Wantai Biological Pharmacy Enterprise Co., Ltd. EV-A71 were all passaged in Vero cells.

### 3.3. Cytotoxicity Assay

The cytotoxic effects of compounds on Vero cells were detected by the cytopathic effect (CPE) assay. Briefly, cells (3 × 10^4^ cells/well) were seeded into 96-well culture plates and incubated overnight. Then, the medium was removed and different concentrations of compounds were applied in triplicate. After 3 days of incubation, the CC_50_ was defined as the concentration that inhibited 50% cellular growth in comparison with the untreated controls, as calculated by the Reed–Muench method.

### 3.4. CPE Inhibition Assay for Anti-EV-A71

The anti-EV-A71 activity of compounds was determined by the CPE inhibition method. Briefly, cells (3 × 10^4^ cells/well) were plated into 96-well culture plates for incubation for 24 h. The medium was then removed and cells were infected with EV-A71 with 100TCID_50_ (50% tissue culture infective doses) in serum-free medium for 1 h at 37 °C. Then, the unbound viruses were removed and various concentrations of compounds were supplemented and incubated for another 48 h. The EC_50_ was determined by the Reed–Muench method. The selectivity index was calculated as the ratio of CC_50_/EC_50_ [15].

### 3.5. Time-of-Addition Assay

The antiviral activity of the tested compound was examined at different time periods prior to, during or after viral infection. Briefly, Vero cells (9 × 10^5^ cells/well) were inoculated with EV-A71 (MOI = 1.0) at 37 °C for 1 h. The media with or without treatment were added during the periods of −24~0 h, −1~0 h, 0~10 h, 1~10 h, 2~10 h, 4~10 h, 6~10 h and 8~10 h. After each incubation period, the collected cells were washed with PBS, and then the viral protein was determined by Western blot.

### 3.6. Western Blot Analysis

Vero cells (9 × 10^5^ cells/well) were plated into 6-well culture plates for incubation for 16 h. The medium was removed and cells were infected with EV-A71 (H, MOI = 0.1). After 1 h, various concentrations of **A3** were supplemented for incubation for another 24 h. The cells were lysed in the M-PER mammalian protein extraction reagent (Thermo, Rockford, IL, USA) containing halt protease inhibitor single-use cocktail (Thermo). The protein concentration was determined by the BCA reagents (Thermo). About 15 μg of proteins was denatured and applied to sodium dodecyl sulfate-polyacrylamide gel electrophoresis (SDS-PAGE). The electrophoresis products were transferred to a polyvinyl idenefluoride (PVDF) film and PVDF membranes were then incubated at room temperature with a specific primary antibody. After a standard washing, membranes were incubated with horse radish peroxidase (HRP)-labeled secondary antibody. The assay was developed using a chemiluminescent substrate. The primary antibodies used in this study included antibodies against β-actin (Cell Signaling Technology), and EV-A71-VP1 (Abnova). Goat anti-mouse HRP-labeled antibodies were obtained from Cell Signaling Technology [15].

### 3.7. Quantitative Reverse-Transcription Polymerase Chain Reaction (qRT-PCR) Quantification

Vero cells (9 × 10^5^cells/well) were plated into 6-well culture plates for incubation for 16 h. The medium was removed and cells were infected with EV-A71 (H, MOI = 0.1). After 1 h, various concentrations of **A3** were supplemented and incubated for another 24 h. The total RNA of the infected cells was extracted using the RNeasy Mini kit (QIAGEN) according to the manufacturer’s instructions. The one-step qRT-PCR was performed with SuperScript III Platinum SYBR Green One-step RT PCR Kit (Invitrogen) using the ABI 7500 Fast Real-Time PCR system (Applied Biosystems). The RNA expression of EV-A71 VP1 was detected with the sense primer 5′-GATATCCCACATTCGGTGA-3′ and the antisense primer 5′-TAGGACACGCTCCATACTCAAG -3′ targeting a conserved region of the VP1 gene. β-Actin mRNA was detected using the sense primers 5′-TGACGGGGT CACCCACA CTGTGCCCATCTA-3′ and the antisense primer 5′-CTAGAAGCATTTG CGGTGGACG ATG-3′. The PCR assay was carried out in a 25 μL volume and the target fragment amplification was carried out as follows: reverse transcription at 50 °C for 3 min; initial activation of HotStar Taq DNA Polymerase at 95 °C for 10 min; 40 cycles in two steps: 95 °C for 15 s, 60 °C for 30 s. The relative amounts of EV-A71 VP1 mRNA were calculated by the comparative Ct method after normalizing the quantity of β-actin [15].

### 3.8. Statistical Analysis

Data are expressed as the mean ± standard error of the mean and were analyzed with one-way ANOVA using MTLAB software (8.6, MathWorks, 2015, Natick, MA, USA). A threshold of *p* < 0.05 was defined as statistically significant.

## 4. Conclusions

In this study, twenty-four 2-aryl-isoindolin-1-one analogs were designed and synthesized using the fragment-hopping strategy. Anti-EV-A71 activity was evaluated in Vero cell cultures infected by different EV-A71 clinical isolates. Ten of the 2-aryl-isoindolin-1-ones showed broad and significant antiviral activity at the submicromolar concentration level. 2-(4-Halophenyl)-isoindolin-1-ones (**A3** and **A4**) exerted more favorable antiviral profiles (SI = 98–199), which is attributed to their significantly lower cytotoxicity as compared to the reference compound pirodavir in four virus strain tests. Preliminary SAR studies suggested that 2-substitutedphenyl-isoindolin-1-one is an important pharmacophore for anti-EV-A71 activity. The small amino group on the isoindolin-1-one moiety was shown to be acceptable for antiviral potency. Lipophilic substitution with size restriction at the para-position of the phenyl group might be beneficial for maintaining antiviral activity. Although the EV-A71 H strain presented a weaker sensitivity to this class of compounds than the other strains, such as BrCr, Shenzhen98 and Jiangsu52, in this study, **A3** treatment significantly decreased the viral VP1 expression at both the protein and RNA levels in a dose-dependent manner. Although the mechanism of action of this class of compounds is still undetermined, the time-of-addition experiment indicated that compound **A3** played a role in the early stage of EV-A71 viral cycle, and presumably acts on the virus entry stage. In summary, 2-aryl-isoindolin-1-one is a promising scaffold with broad anti-EV-A71 activity for further study. On-going studies include detailed SAR, the mechanism of action and in vivo evaluation of compound **A3** in an animal model.

## Figures and Tables

**Table 1:** Antiviral activity of tested compounds towards four enterovirus A71 (EV-A71) clinical isolates in vitro.

Compd.	CC_50_ (µM)	BrCr	SI	H	SI	Shenzhen98	SI	Jiangsu52	SI
		EC_50_ (µM)		EC_50_ (µM)		EC_50_ (µM)		EC_50_ (µM)	
**A1**	370.14 ± 39.81	7.87 ± 0.91	47	28.71 ± 4.07	13	7.87 ± 0.91	47	7.87 ± 0.91	47
**A2**	218.06 ± 22.31	3.23 ± 0.25	67	4.43 ± 0.88	49	4.43 ± 0.68	49	3.74 ± 0.80	58
**A3**	257.43 ± 35.33	1.29 ± 0.24	199	1.53 ± 0.16	168	1.29 ± 0.24	199	1.29 ± 0.24	199
**A4**	172.64 ± 34.05	1.23 ± 0.28	140	1.76 ± 0.19	98	1.76 ± 0.15	98	1.32 ± 0.22	131
**A5**	68.39 ± 5.72	1.27 ± 0.01	54	1.81 ± 0.21	38	1.39 ± 0.17	49	1.27 ± 0.01	54
**A6**	218.06 ± 22.31	NA *	/	NA	/	NA	/	NA	/
**A7**	318.23 ± 54.51	7.27 ± 0.86	44	9.35 ± 1.44	34	7.27 ± 0.86	44	7.92 ± 0.86	40
**A8**	76.50 ± 13.10	12.62 ± 2.22	/	NA	/	12.95 ± 2.00	/	12.62 ± 2.22	/
**A9**	96.47 ± 9.34	17.00 ± 2.92	/	NA	/	17.00 ± 2.92	/	17.00 ± 2.92	/
**A10**	46.22	12.92 ± 2.24	/	13.65 ± 5.71	/	14.61 ± 2.24	/	14.61 ± 2.24	/
**A11**	115.47	NA	/	NA	/	NA	/	NA	/
**A12**	179.56 ± 27.26	26.99 ± 3.38	/	24.44 ± 9.37	/	30.26 ± 2.38	/	30.26 ± 2.38	/
**A13**	38.49	>22.22	/	>22.22	/	>22.22	/	>22.22	/
**4**	230.94	5.27 ± 0.44	44	11.66 ± 2.10	19	5.27 ± 0.44	43	5.74 ± 0.54	40
**5**	158.98 ± 17.10	9.12 ± 0.76	17	16.24 ± 1.90	9	9.12 ± 0.76	17	9.70 ± 0.76	16
**6**	172.64 ± 34.05	1.89 ± 0.32	91	2.11 ± 0.23	82	2.00 ± 0.25	86	1.89 ± 0.32	91
**B1**	68.39 ± 5.72	1.59 ± 0.35	43	5.41 ± 0.63	13	1.59 ± 0.35	43	1.59 ± 0.43	43
**B2**	257.43 ± 35.33	34.63 ± 6.54	/	NA	/	34.63 ± 6.54	/	40.28 ± 5.54	/
**B3**	230.94 ± 0.00	18.42 ± 2.41	13	NA	/	18.42 ± 2.41	13	20.61 ± 1.01	11
**B4**	123.89 ± 12.59	NA	/	NA	/	NA	/	NA	/
**C1**	151.55 ± 27.01	5.85 ± 0.60	26	>44.44	/	5.85 ± 0.60	26	5.91 ± 0.65	25
**C2**	230.94 ± 0.00	NA	/	NA	/	NA	/	NA	/
**C3**	113.42 ± 17.24	NA	/	NA	/	NA	/	NA	/
**D1**	115.47	NA	/	66.67	/	NA	/	56.45 ± 14.45	2
**D2**	115.47	19.56 ± 3.77	/	19.56 ± 3.77	/	19.81 ± 3.40	/	15.12 ± 3.23	/
**D3**	42.35 ± 5.47	2.71 ± 0.35	16	2.09 ± 0.54	20	3.73 ± 0.78	11	1.29 ± 0.33	32
**pirodavir**	33.33	0.38 ± 0.04	88	0.76 ± 0.09	44	0.50 ± 0.06	67	0.62 ± 0.10	54

* NA means no antiviral activity was observed at maximum nontoxic concentration; / represents selectivity index (SI) value of less than 10.
